# Ethical Issues Encountered by Nurse Managers Working With Older Adults in Long-Term Care Settings: A Qualitative Interview Study

**DOI:** 10.1155/jonm/3978256

**Published:** 2025-05-14

**Authors:** Anna-Liisa Arjama, Riitta Suhonen, Mari Kangasniemi

**Affiliations:** ^1^Department of Nursing Science, University of Turku, Turku 20014, Finland; ^2^Turku University Hospital, Wellbeing Services County of Southwest Finland, Turku, Finland

## Abstract

**Introduction:** Nurse managers (NM) face ongoing ethical issues when they work with older adults in long-term care settings (LTCS), including around end-of-life care. Legislation and healthcare ethics guide the provision of ethical care to older adults in a changing societal and global context.

**Research Aim:** Our aim was to describe the ethical issues encountered by NMs.

**Methods:** This qualitative study involved 23 NMs from seven randomly selected organisations who participated in semistructured focus group interviews in 2021. We analysed the data using inductive content analysis.

**Ethical Considerations:** The subject of this study was sensitive and reflected the participants' individual views. They provided informed consent and their anonymity was guaranteed.

**Results:** Ethical issues faced by NMs are related to residents' rights to self-determination, ethical decision-making about staff and procedures, providing ethical leadership despite having conflicting roles, and defending ethics in LTCS on a societal level. NMs struggled to spend sufficient time supporting their staff during everyday care.

**Conclusions:** The ethical issues encountered by NMs are multidimensional and have both external and internal causes. NMs often deal with ethical issues on their own. Structuring their roles so that they can focus more on daily care could help NMs to provide more effective leadership and get more involved in their organisation's decision-making. Further research into the impact of NMs' backgrounds on their performance and responsibilities could provide new insight which would be useful in educating NMs and designing relevant organisational structures.

**Implications for Nursing Management:** Our research can be applied to practice, policy, education and research.

**Practice:** NMs' daily work should be organised in a way that enables them to work closely with residents and staff. Reducing their secondary tasks could increase the time available to manage staff and provide individual coaching for those with different independent abilities. Being present during daily care would make it easier to deal with ethical issues in a timely manner, which could reduce staff's moral distress and increase their well-being at work.

**Policy:** Understanding the importance of the role of NMs could help policy makers in planning LTCS care. Involving NMs in decision-making in organisations and society could increase awareness about the relevant ethical issues and improve the care that residents receive and the well-being of staff and new members. For example, how many employees a single NM can manage could be defined in the same way as the number of staff per resident has been determined. Implementing ethics committees in LTCS could provide a mechanism for considering the views of NMs.

**Education:** Ethical issues in the care of older adults should be included in the curriculum of those studying for the profession. Ethical issues change over time as the world around them changes. Addressing ethical issues should be a continuous theme in continuing education for relevant workers.

**Research:** This qualitative study gave a voice to LTCS NMs in a society with a rapidly ageing population and labour shortages. Ethical issues faced by NMs were related to implementation of nursing and healthcare values in the daily care of residents. In the future, generalisable knowledge is needed about what is the ethical climate in LTCS workplaces, and what is the role of NMs' and care workers' ethical competencies and attitudes towards ageing when performing daily care in the LTCS. In addition, it is noteworthy that the ethical issues' NMs faced were related to policy decisions made in the surrounding society, and NMs felt they had no power or ability to influence on them. In the future, more knowledge is needed to understand how NMs in LTCS, but also in other areas of nursing, identify and consider their role in ethical issues related to health policy.

## 1. Introduction

In long-term care settings (LTCS) for older adults, nurse managers (NMs) are responsible for promoting ethical values including residents' independence, self-determination and dignity [1, 2] under the guidance of legislation, healthcare ethics and professional ethics. Older adults who require assistance due to diminished functional capacity have the right to access affordable, high-quality care. The principle of LTCS is to offer a home-like environment even though residents may face multiple health issues, be frail and depend on others for everyday assistance [3]. Residents are supposed to receive holistic, person-centred care based on the ethical values of beneficence and avoiding harm [4, 5]. While nursing home employees are responsible for residents, family members are also involved in their lives, and the care relationship is a close on. It also inevitably ends with death [6–10]. NMs have an impact on both the well-being of residents and other staff, and the success of the organisation as a whole [11, 12]. Ethical issues thus pervade everyday situations in LTCS, and relevant decisions about good and bad, right and wrong, must be continually reflected upon [13].

NMs in LTCS for older adults face numerous ethical issues in their daily work. The more closely they work with residents, the more closely the ethical issues they face revolve around everyday decisions. Front-line managers are responsible for residents' daily care, but other parties involved in the care, for example, family members, may completely disagree with them [1, 11–13]. Front-line NMs also play a key role in realising and transmitting fundamental values to the staff they manage. They use ethical argumentation, reflection and decision-making to align staff with their organisation's values and alleviate the moral distress that often arises in caring for older adults [14–16]. Moral distress occurs in situations where professionals cannot do their job as well as they would like, usually due to scarce resources and the need to prioritise the type of care to be provided. If moral distress persists for a long time or it is not possible to discuss it with a work group or supervisor, it can damage work well-being and stimulate the desire to change jobs [17, 18]. With the support and collaboration of higher-level of management, front-line NMs are responsible for facilitating and implementing relevant guidelines and policies in their unit [19, 20]. Ethical issues can also arise from matters outside the organisation, such as ongoing societal and global changes that shape NMs' work and bring new ethical issues to the fore. The society expects NMs at all management levels to have insight and commitment, and be assertive when making decisions in ethically challenging situations [16, 21, 22].

Ethical issues in long-term care are likely to becoming increasingly pressing as population in Western countries age and suitable labour becomes scarce. In contrast to other healthcare settings, most employees in LTCS are educated to a secondary or lower level, meaning that they have been taught little about ethics despite working independently with vulnerable residents with multiple health conditions [23]. Societally, LTCS work has a reputation for being challenging work, which may affect team cohesion, for example, by causing a decline in ethical climate. Ethical climate refers to various behaviours and circumstances that affect the handling and management of ethical issues [15, 24, 25]. In addition, the COVID-19 pandemic challenged the ethical assessment of NMs in many ways. Residents' rights had to be restricted, and staff had to learn a new way of working with them [26, 27].

Previous research into ethical issues from NMs' point of view has drawn on various care environments, including hospitals, primary care and home care [8, 15, 19, 28–30]. This study focuses particularly on how NMs in front-line and middle management position themselves experience ethical issues in their work.

The aim of this study was to describe the types of ethical issue that NMs encounter in their work. The results may be useful to supporting, educating and training NMs and further developing LTCS services.

## 2. Methods

### 2.1. Design

This qualitative interview study is based on semistructured focus group interviews with 23 NMs in Finland, carried out in 2021. We used inductive content analysis to describe and conceptualise the ethical issues that NMs encounter with older adults in LTCS [31].

### 2.2. Study Setting and Recruitment

In Finland, municipalities are responsible for providing LTCS for older adults directly or through private service providers [32]. LTCS for older adults effectively combines social and health care. It is considered a social service within which nursing care, representing health care (e.g., medication management), is integrated into daily care. Staff working in LTCS is required to have both social and healthcare qualifications if they are involved in administering medication [33]. LTCS is provided to older adults whose functional capacity means they require 24 h assistance. During the data collection period in 2021, social and healthcare services in Finland were preparing for the implementation of reforms in 2024, which would restructure 300 municipalities into 23 county-based wellbeing services, improve patient centredness and access to care and control costs [34]. In 2021, 1730 units provided 24 h long-term care for older adults [35], with a total of 39,000 nursing care professionals [36]. NMs at front-line and middle management levels in LTCS usually have a bachelor's or master's degree in health or social work. This equates to levels 6-7 in the European qualification framework (EQF), an eight-level structure of core competencies across Europe [37].

We recruited participants using a national register of LTCS facilities [35]. We randomly sampled eight of the 1730 registered units using an electronic randomiser [38]. Seven of the eight organisations were willing to participate and granted permission for the research. These organisations were in different parts of Finland: one in a metropolitan area, one in a big city, two in average sized cities and three in rural areas. Four of the organisations were public, and three were private providers. One researcher (A-LA) contacted the organisations to recruit NMs. All NMs from the participating units were invited to be interviewed. We do not know how many invitees did not participate in the interviews. Interviews were carried out at NMs' workplaces, in separate, quiet meeting rooms. With their employers' permission, NMs were interviewed during their working hours. They were not directly remunerated for participating in the interview but received their salary for the time involved.

### 2.3. Participants' Characteristics

Our target group was front-line and middle managers working with older adults in LTCS. Altogether, 23 NMs participated in nine interviews which were conducted either face-to-face, remotely, or in a hybrid format, according to participants' wishes (Table 1). Most (19 of 23) worked in the public sector (Table 1). We strove to convene larger groups but, due to the small size of the participating organisations and units, we had eight group interviews with 2–4 participants and one individual interview (Table 1).

Nineteen participants were registered nurses (RNs) (EQF 6), and for seven of them, this was their highest degree. Six had graduated from a university of applied sciences with a Master's in Health Care (EQF 7). Five had graduated from university with a Master's in Health Sciences (EQF 7). One participant had a Bachelor's Degree in Social work (EQF 7), one a Degree in Administrative Sciences (EQF 7), one a Degree in Social Sciences (EQF 7) and one a Master's Degree in Physiotherapy (EQF 7).

Participants' mean age was 50 years (range 31–64), and 13 of the 23 had worked in older adults' services for 10–30 years. Almost half of participants had participated in supplementary skills education on ethics after their degree, usually over the course of a few days. Two NMs had participated in supplementary skills education related to end-of-life care, one on using humour in nursing and one on ethical principles. One NM had participated in a discussion group on workplace ethics.

### 2.4. Data Collection

We used a semistructured interview guide [39] developed on the basis of the existing literature on ethical issues and LTCS and addressing two areas of enquiry (Table 2). The first concerned ethical issues in daily care during the COVID-19 pandemic [40, 41], and the second ethical issues relating to providing residents with comprehensive care and cooperating with families [6, 9, 10]. The guide also covered working as a professional in LTCS [18, 42], management and organisation [43], and how LTCS is perceived by society [15, 24]. We asked probing questions to deepen our understanding of the issues raised by participants [44]. We tested the interview guide through a pilot interview with one NM. This showed that the interview guide did not need to be altered and helped us to decide how much time to dedicate to each area of enquiry. The pilot interview was not included in the data [39].

We chose to gather data through focus group interviews so that we could understand participants' experiences and beliefs [45] about ethical issues in LTCS as a shared phenomenon in NMs' daily practice. In groups and pairs, participants were able to discuss the issues together and make comments that they may not have made during individual interviews [46]. The interviews generated knowledge about the meanings behind participants' views [45], and they had a free-flowing discussion in a relaxed environment [46]. To reduce the disadvantages of the focus group method, such as discussing issues in ways that the interviewer is presumed to want to hear, participants were told that there were no right or wrong answers and that the questions were not intended to assess their success at work [47]. The confidentiality of the discussions was emphasised, and participants were encouraged to bring different opinions and comment on each others' views to better understand ethical issues. No participant expressed any distress during an interview. Rather, the lengthiness of the interviews reflected participants' high levels commitment, and they felt that the discussions were almost like job counselling sessions. No conflicts arose between participants, and they did not feel the need to take breaks. One researcher (A-LA) conducted and recorded all of the interviews. The researcher had a background as a NM and was very familiar with the subject, enabling them to ask questions that were appropriate to everyday work [48].

### 2.5. Data Analysis: Inductive Content Analysis

We recorded the interviews, which each lasted for between 100–120 min and produced 17 h of material. We used inductive content analysis, and deriving coding categories were directly from the data [31]. First, the researcher who had carried out the interviews (A-LA) transcribed the recordings manually and verbatim shortly after the interviews. We read these novel data several times to grasp its meaning and then word by word to derive meaning units from the sentences, considering their literal manifest and deeper content. NVivo software was used for coding [49]. Then, we took notes on the issues, thoughts and initial analysis emerging from the text. We identified 283 meaning units, sorted these into categories, and then organised and grouped them. All of the authors took part, combining the categories into subthemes and then themes, and naming them inductively (Table 3). This phase was performed without software and took several weeks. We integrated the issues relating to the COVID-19 pandemic into the other themes because they were discussed in connection with other generic issues.

### 2.6. Ethical Considerations

The principles of research ethics were followed throughout the study process [50]. According to Finnish legislation, a study of this type with legally competent adults does not require ethical approval [51]. Rather, a research permit was obtained from each participating organisation, following their own approval processes. The primary ethical consideration was confidential participation.

Participants provided their written, informed consent to participation and recording the interviews. The subject of this study was sensitive and reflected participants' individual views. We assured participants that their participation would be confidential and the data anonymised so that individual participants could not be identified from the results. Participants were also able to withdraw their participation at any time without consequences, and only the researchers had access to the data. The data were stored in a password-protected computer [50].

### 2.7. Rigour and Reflexivity

The trustworthiness of this study was assessed using four criteria: credibility, dependability, confirmability and transferability [52]. We ensured credibility by using an interview guide based on previous literature [39] and continued the interviews until we reached data saturation. Participants were able to discuss and share perceptions about similar daily management situations. The exception was a single participant who was interviewed separately for practical reasons. To improve the dependability of the study, the authors worked together to compare the categories, transcribed the text throughout the analysis and discussed each step until a consensus was reached. To ensure confirmability, we included some of the original expressions in the findings, to illustrate how participants felt and expressed themselves during the interviews. We also described how we created the main themes from the original expression and then used them to create meaning units, codes, subthemes and themes. Transferability was ensured by describing the data collection method and analysis in detail. We collected background information from participants and explained the research environment to clarify the context of this study [53, 54]. This study focuses on ethical issues in LTCS for older adults, but its results reflect the principles of health care ethics more generally. This means that they could be applied to other areas of public service where a trained manager is responsible for providing care.

## 3. Results

The ethical issues encountered by the NMs were related to ensuring residents' self-determination, the NMs' role in the organisation and ethical decision-making about staff and procedures and defending ethical care in LTCS (Table 4).

### 3.1. Theme I: Ensuring Residents' Right to Self-Determination

The NMs emphasised the importance of being around when daily care was being provided, so that they could identify ethically challenging situations. This included ensuring the residents' rights to self-determination. They needed to be aware of the decisions made about the residents' care and being familiar with the residents and their family members so that they could ensure they received person-centred care.

The NMs were advocated for both residents and staff and this meant that they needed to observe practical situations closely to provide fair guidance. They believed that staff needed support to provide person-centred care to prevent situations where, for example, the staff would restrict resident's movement due to rush. The NMs thought it was important for residents to continue their individual routines, even when they lived in LTCS. They thought that residents were not supposed to be easy and quiet and that they needed to be able to express their emotions and feel close to people. NMs trusted the professionality of the staff, but also monitored their actions to ensure they provided ethical high-quality care. Sometimes NMs intervened in situations if this was lacking, such as when the staff's behaviour exacerbated the residents' symptoms.

‘With some staff it's easy but some don't understand that it's their own behaviour that triggers the resident's behavioural symptoms.' (RN, front-line NM, private sector, 22 years working for older adults' services)

The NMs wanted to be aware of the decisions that the staff had made about the residents' care. They wanted to know how the staff justified decisions about daily care, for example, if the residents and/or their family members disagreed with the decisions made by staff. This could include basic decisions about necessary medication or nutrition. In such cases, they needed to talk with the different parties. The NMs also wanted to know what information staff shared with the doctor, because the staffs' perceptions of the residents' well-being varied and the doctors made decisions based on what they told them. NMs monitored the staff's decision-making in daily situations and what they had written in the patient information system.

‘Sometimes when I read the entries in patient information system, I don't even understand what this employee has written.' (RN, front-line NM, private sector, 14 years working for older adults' services)

NMs also wanted to get to know the residents and their family to ensure that the resident received person-centred care. The NMs thought that involving them in decision-making ensured that the resident was not just a passive object who received treatment. The NMs spoke about situations where staff were afraid of engaging with family members and needed their encouragement to do so. For example, the staff were afraid to intervene when family did not maintain safe distances during the pandemic. They were also afraid of feedback or that talking with family members would take too much time. The NMs had to ensure that residents and family members received proper information about residents' care. For example, the decision not to restrict a resident's movement when there was a risk of falling had to be justified to family members. Also, the possibility of end-of-life care had to be shared in detail and repeatedly.

‘We have made it clear to family members that we want the residents to move around independently. We have also reminded them that there is a risk that the residents could fall and be injured and have asked family members to provide padded hip pants.' (Master of Health Care, front-line NM, private sector, 2 years working for older adults' services)

### 3.2. Theme II: Ethical Decision-Making About Staff and Procedures

The NMs described their ethical decision-making about staff and unit procedures. They said that they had to prioritise their daily responsibilities, by treating staff equally, but also meeting their individual needs. They also needed to maintain their ability to make ethical decisions even when instructions were incomplete and changed rapidly.

Most NMs did not have enough time to discharge all their responsibilities. They were committed to their roles, but felt their workloads were unfair. The NMs knew that prioritising their responsibilities had a direct impact on numerous stakeholders. For example, they were expected to attend meetings organised by their employer, but then they had no time for proactive measures that could have eased the workload for the coming days. Also, if they allocated their time to one employee, resident or family member, they felt that all their other tasks were neglected. They felt it was unfair that they had to balance their time between taking care of the staffs' occupational well-being, and their own, and that their work balance was affected by continuously working overtime. NMs also felt it hard to take days off and, if they did, tasks accumulated that further increased their sense of burden.

‘When I came back from vacation, I came to work at 8am in the morning and left here at midnight. This went on for many days. After that week, I almost cried when a family member filed a complaint about the care given to a resident.' (Master of Administrative Sciences, middle management, public sector, 15 years working for older adults' services)

The NMS had to find the balance between treating their staff equally, but also meeting their individual needs for support and coaching, because they had different capabilities. Staff shortage in LTCS meant organisations hired staff they would previously have excluded due to a lack of professional skills. The organisations expected the NMs to offer individual coaching for staff, but the NMs had to compromise as there were so many inexperienced workers. The NMs thought that every employee should contribute to providing residents with good care. NMs adapted work to suite the staff member's skills. They thought that staff should be able to work without worrying about the negative effect on their well-being. For example, indoor air problems caused by mould and extra pressure on permanent staff were common problems. NMs believed that nonmanagerial staff should have the right to just be responsible for their own work performance. The NMs felt that ethical issues were part of care work and sometimes staff had to act against their own ethical values, for example, if there was a rush caused by a sudden event. However, the NMs said that these situations should be exceptional and not last long. The NMs agreed that responding to the needs of their staff in a timely manner would prevent problems from accumulating or growing. For example, it was crucial to resolve any conflict among staff or with family members quickly, because unresolved conflicts lead to sick leave.

‘Having different cultures in the working team often provides challenges for my work. It is my responsibility to find out what is causing friction between staff and how to solve it.' (Master of Health Sciences, front-line NM, public sector 23 years working experience in older adults' services)

The NMs said they had to maintain their ethical decision-making ability, even when instructions were incomplete or rapidly changing. It was hard to follow and implement unclear instructions, particularly during the COVID-19 pandemic, but NMs were still responsible for ensuring that their staff followed the latest instructions. This was difficult when the staff worked three shifts, and there could be daily changes during the pandemic. Sometimes, the instructions conflicted, and the NMs had to choose which to follow. For example, if qualified substitutes were not available, they had to decide whether it was best to have fewer staff caring for residents or employ someone who was not competent for the role.

‘This is a big organisation and there are a shocking number of instructions. It is not possible to be clear about which instruction to follow.' (RN, front-line NM, public sector, 10 years working for older adults' services)

### 3.3. Theme III: Providing Ethical Leadership Despite Conflicting Roles

NMs aimed to provide ethical leadership but had conflicting roles in their organisations. Whether they successfully handled ethically challenging situation depended on how much support they received from their supervisors and senior management. It also depended on how much time they had to spend on secondary tasks, and their obligations to their employer, even if it was against their conscience.

According to the NMs, receiving support in ethically challenging situations from a supervisor or senior management affected how well they could act ethically. They felt that they lacked support if they could not reach their supervisor by phone or e-mail or if their supervisors did not have enough time to discuss daily challenges. They also felt that their employer kept adding tasks without considering the existing workload. However, even when the NMs were not satisfied with the support they received, they felt the freedom of their work compensated them for the burden and they did not want to complain. Also, the fear of being criticised or facing consequences often made them conceal challenging issues. NMs felt it was unfair that they were reprimanded by their superiors if they missed less important tasks, such as statistics, or because they had prioritised other, more important tasks. However, the fear of consequences was reduced when the NMs had a trusting relationship with their supervisor or senior managers. This meant that they were able to defend their ethical principles, even if they conflicted with the employer's instructions. Sometimes, the NMs found the expectations of senior management unrealistic and did not understand why they wanted to implement changes. The NMs said the reforms were rushed, but not monitored or evaluated afterwards. They disapproved of the fact that daily good care was suddenly pushed aside, and senior managers were busy concentrating on one topic. NMs said that they needed more time to implement changes because they involved vulnerable residents and needed to be communicated to staff working on three shifts.

‘If I have a question for my manager, it is wise to email her so that I can get a written answer that I can refer to later.' (RN, front-line NM, public sector, 30 years working for older adults' services)

The NMs said that a large part of their time is spent on work that did not require their skills or seniority. These secondary tasks, together with misaligned support services, wasted time that should have spent on ethically important matters, such as supporting staff and developing quality of care. One NM could have dozens of staff with exceptional situations that required workplace adjustment, such as mental health problems or small children. There were issues that could not be dealt by occupational health care or human resources and activities, such as regular discussions or working time arrangements. In some organisations, half of NMs' working time was calculated to be used in care work. The NMs felt that this is not fair, because practically all their working time was spent on administrative work, and they did not have time to help care for the residents. If they did take part in caring for residents, their work was constantly interrupted by the phone ringing or administrative tasks piled up because they were busy with resident work.

‘The staff consider their own affairs important and do not understand that I must spend time on administrative work. The senior management does not understand that I am needed to support daily work outside the office.' (Master of Health Sciences, front-line NM, public sector, 6 years working for older adults' services)

The NMs also discussed their obligations to the employer even if it conflicted with their conscience. They knew that safety of staff and residents could not be guaranteed in all situations, and this weighed them down. For example, their current premises would make evacuating residents impossible if there was fire, and they did not have enough staff at night to observe residents. They also felt bad if they promised family members individual care for a resident, knowing it could not happen due to staffing shortages.

‘Sometimes I wonder if the senior management could loosen up a bit, so that we could focus on providing good care to the residents.' (RN, front-line NM, public sector, 22 years working for older adults' services)

### 3.4. Theme IV: Defending Ethics in LTCS on Society Level

The NMs described how they defended ethics in LTCS when they had to deal with society without the resources they needed. They highlighted the importance of self-compassion and were aware that they needed to maintain high standards despite poor resources. The NMs also felt that there was conflict between regulations and daily care needs.

The NMs said that they often faced accusations about the ethical consequences of labour shortages in LTCS, but they felt self-compassion for themselves, because they knew that they were not is position to solve them. Instead, they thought that the core ethical problems relied on decisions made in society. NMs thought that society was not adequately prepared for the ageing population. Salaries were unfairly low and not enough to cover the cost of living, and older adults did not receive the care they needed. They were worried that labour shortages had already weakened older adults' access to the care facility they worked in. However, they felt that they were in a better position than their home care colleagues who cared for clients with advanced memory issues who would eventually need nursing home care.

‘A couple of staff have quit this week. They have moved to a smaller town, where the cost of living is lower, because the employee's salary is not enough to live in the capital region.' (Master of Social Sciences, middle management, public sector, 14 years working for older adults' services)

The NMs discussed how it felt to manage poorly resourced services to high standards. They described lofty goals in terms of treatment but pointed put they could not even guarantee decent medical services for residents. These endangered residents care and increased the ethical burden on staff when they did not get help they needed. NMs also criticised the fact that older adults or staff were not included in planning services. This meant that the residents were either overtreated like hospital patients or undertreated because did not receive the services they needed. The NMs were worried that there were insufficient discussions in society about end-of-life, which was seen as an expensive service rather than one that provided what was best for individuals.

‘Society demands the kind of care that our resources cannot provide' (Master of Social Sciences, middle management, public sector, 14 years working for older adults' services)

NMs described their need for ethical balance between LTCS regulation and practical needs. In their opinion, statistics and monitoring took an unreasonable amount of their working time but could not achieve the well-being of residents or staff. The NMs felt that the number of statistics and monitoring required had increased in recent years, but they did not know who benefited from the information. For example, they understood staff numbers needed to be increased but recruitment took an inordinate amount of time, and they were forced to recruit incompetent people to make up the numbers. Even small things, such as the fact that the night fast could not exceed 11 h, made it difficult to implement individual treatment, as some residents wanted to sleep longer. There were also conflicting regulations. For example, NMs were not allowed to ask staff whether they were vaccinated against COVID-19, but they had to ensure that only vaccinated staff take care of residents with symptoms. The NMs had experienced it all. Some staff had lied about being vaccinated to avoid taking care of residents with COVID-19. In some organisations, staff had vaccinated each other, all in same room, compromising data protection. The regulations for LTCS were considered strict, and the NMs tried to follow them, but they also found that they were unclear and could be interpreted in different ways. For example, in some units, the doors had to be closed even though the residents wanted to look out the corridor. In other units, it was strictly forbidden to lock doors, even though other residents entered rooms and scared the occupants.

‘Every now and then I wish that long-term care for older adults could be rebuilt from scratch. We would abandon the current, unproper regulations and invest in good care.' (Master of Social Sciences, middle management, public sector, 14 years working for older adults' services)

## 4. Discussion

The ethical issues faced by NMs in their work relate to residents' right to self-determination despite needing significant assistance; making daily decisions about the care provided by staff; and conflicting role between residents, staff and senior management. NMs are responsible for staff who work independently with frail residents. Most of the staff have a secondary education, and decisions about how to care for residents are based on their judgement. This differs significantly from, for example, specialist nursing work, where most employees have a higher education and are able to consult a physician 24 h a day [15, 55].

### 4.1. NMs as Advocates for Residents' Right to Self-Determination in Society

Maintaining residents' self-determination in LTCS is essential to realising their human dignity and is an important value in nursing care. However, it is constantly challenging due to factors both within and beyond LTCS facilities.

In our study, societal values and global changes were reflected in NMs' work. For example, behind staff shortages lay sector-wide decisions about older adults' care and working conditions, NMs were concerned that staff shortages led to staff moving between departments and prioritising some aspects of care over others, would compromise the implementation of person-centred care [56]. Labour shortage means that NMs are forced to hire people who do not necessarily have sufficient skills to do nursing work or the desire to work in LTCS. According to previous studies, a lack of ethical competence—awareness about the law, principles and values guiding their work and capacity for ethical reflection, decision-making, and action—can cause moral distress for staff if they are left alone in difficult situations involving residents' dignity or the quality of end-of-life care. If staff cannot manage this distress, their job satisfaction may decrease, and they may consider changing jobs [15, 23]. However, where NMs are able to interact with residents, their family members and other staff, this enables NMs to develop greater insight and support for dealing with ethical issues [57, 58]. In our study, NMs pointed out the importance of timely and regular discussions with staff about the ethics of daily care situations. They found that ethical reflection can help NMs to justify necessary changes, encourage staff to practice person-centred care, and confront family members during challenging situations [23]. However, NMs' responsibilities were so extensive that it was not possible for them to be present during daily care situations, observing the implementation of care and providing support to staff.

Since this study began, the Finnish government has changed, and earlier initiatives in favour of increasing staffing for LTCS have been shelved in favour of making economic savings. Nonetheless, the standards for providing care to older adults have become stricter over recent decades. Staffing levels and skills requirements are regulated by law [3], and residents' functional capacity is monitored, using precise tools. This means that staff need ICT skills as well as nursing skills, and spend more time on computers rather than with residents. [59]. This means that staff need ICT skills as well as nursing skills, and spend more time on computers rather than with residents. The work-related responsibilities of particular professional groups have also been defined by legislation. For example, participating in medical treatment requires a degree in social and health care and a valid medical treatment permit [33]. Other rapid global changes to which NMs have had to adapt include the COVID-19 pandemic. During the pandemic, the individual needs of residents were neglected to protect the group and society [27, 40]. According to our data, NMs had to make difficult, independent decisions based on insufficient instructions. NMs felt that society thought that problems in LTCS for older adults could be solved with good leadership. However, according to our participants, resolving such problems rely on the entire management structure of organisations, not front-line or middle managers who rarely have decision-making power. NMs also felt that society had tried to secure the well-being of older adults but created structures that put a burden on NMs with no corresponding benefit.

### 4.2. NMs' Capacity and Insufficiency: Constant Decision-Making in a Conflicting Role

Day to day, NMs work is situated between residents, family members, staff and senior management. In this work, ethical reflection mainly arises around everyday events, but it is underpinned by established ethical values and principles [2, 4]. Our study found that the same ethical values could be implemented through different actions depending on the level at which decisions are made.

We found that NMs need nursing skills to be able to continuously prioritise their tasks and ensure that their work can be carried out in compliance with legislation, healthcare ethics and professional ethics. NMs all had degrees in social and health care, although their backgrounds were diverse. In Finland, NMs need an educational background in health and social care [3] and their staff expect them to have nursing skills so that they can support them in making decisions about residents' daily care [60]. While their educational background ensures that NMs can provide credible support to their staff on ethical issues relating to daily care, they may not see this as an opportunity to support the employee rather than resolving the issue themselves [8]. However, NMs have an important role in supporting their staff and their behaviour has an impact on how staff implement shared values in their daily work [8, 28, 61]. Conceptualising ethics may be challenging because NMs do not necessarily have the skills or time for ethical discussions. Also, reflecting on ethical issues may evoke negative emotions in NMs, and recognising these emotions is a prerequisite to them carrying out ethical reflection with their staff [61]. The staff need to be encouraged and motivated to take part [8, 14, 62]. According to our results, NMs realise the importance of their roles, and both want and were expected to use their time to provide staff with individual coaching. Decisions about residents' well-being can be made by individual employees, including temporary staff or students with limited skills, without the NM being present or intervening. However, this might increase moral distress for all concerned because poor decisions have long term impacts in LTCS [17, 18]. Further research into the impact of NMs' backgrounds on their performance could provide insight that would be useful in shaping NMs' continuous education.

In our study, NMs hoped that their senior managers would recognise the ethical issues relating to LTCS work and take these into account when proposing changes or making decisions. Staff members focus on the care that residents receive and securing their own well-being at work [1, 7], but organisational structures may hinder them from making decisions and force them to compromise basic nursing values [63]. Finnish law states that LTCS nursing homes for older adults should provide a homely environment so that residents feel their lives have meaning and do not feel lonely [3]. Person-centred care is known to be an indicator of high-quality care in LTCS, particularly in dementia care. An organisation's entire operations should support the implementation of person-centred care [56]. However, our findings show that the proposals developed by senior managers do not steer services in the direction that NMs hope they will. Rather, they caused ethical conflict by increasing the workload of permanent staff. It seems that senior managers try to lighten NMs' workload by providing support services, but NMs want to participate more closely in the process so that ethical issues could be considered during decision-making. Our study confirms that, despite these issues, NMs are satisfied with their work because of their perceived autonomy and power to affect things positively [64, 65]. NMs are motivated to influence decisions so that frail residents receive high-quality care and staff enjoy their work. Further research into the issues that front-line NMs have the power to influence and the matters for which they are responsible may provide valuable insight for the further development of care for older adults and the design of organisational structures.

### 4.3. Strengths and Limitations

The strength of this study is that we provided NMs with the chance to take part in interviews remotely or face-to-face, overcoming long distances that could have been a barrier to participation [46]. This ensured the richness of the data, made participation easier and helped to create an atmosphere of trust during the interviews. We collected data until we reached saturation [45]. Focus group interviews were an appropriate method for gathering data as the research topic was complex and previous knowledge was scarce. There were a few limitations to this study. Pilot testing the interview guide with more participants in a focus group would have better reflected the actual interview situation. Possible biases related to focus groups, such as groupthink, social desirability bias and response set bias, were mitigated by carefully informing participants and asking each participant in turn to answer questions. Participants were also informed about the interviewer's relevant background to encourage them to give practical examples from their work [47, 48]. One participant could not be interviewed with others and missed the benefits of being in a group interview. However, the content provided by the participant was still valuable [45]. More information about the content and duration of skills education that NMs had participated in during their working lives would have been informative. Information on the number of NMs in Finland could help us to understand how widespread ethical issues in LTCS are, but there is no information on how many NMs there are in Finland. To mitigate the possible limitation of one-sided text interpretation, all authors participated in completing the analysis [31].

## 5. Conclusions

In LTCS for older adults, the ethical issues encountered by NMs relate to residents' self-determination from an individual and societal perspective and NMs' constant decision-making in conflicting roles. Self-determination for residents in LTCS for older adults is threatened by distant management, organisational structures, and societal values and changes. From the societal perspective, NMs in LTCS are in a responsible position as the number of older adults is increasing and society is making concessions about the quality of LTCS.

This study emphasised the multidimensional nature of ethical issues in LTCS from the perspective of NMs. LTCS facilities constitute a challenging operating environment due to residents' frailty and staffs' independence in caring for them. NMs need to handle fundamental questions from staff, residents and their families. NMs working in LTCS must rely on their own personal ethics, and it can be difficult for them to manage the whole spectrum from an individual's initial admission into care through to their death. NMs in front-line or middle management are the visible parts of the organisation as they have direct connections to both senior management and residents. Enabling NMs to spend more time immersed in daily care could support them to lead their staff and be involved in organisational decision-making. Senior managers should also be committed to ethical care, not just front-line and middle management, who rarely have the power to influence an organisation's operations. Further interdisciplinary research into the organisation of care in LCTS could generate useful insights to support the reform of relevant services.

## Figures and Tables

**Table 1:** Participant demographics.

Group	Format	Sector	NMs (*n* = 23)	Highest degree/(EQF level)	Work experience in years
1	Combination of remote and face-to-face	Public	3	Registered nurse (6)	30
				Bachelor of social services (6)	9
				Master of health care (7)	5

2	Face-to-face	Public	4	Master of administrative sciences (7)	15
				Master of health sciences (7)	23
				Master of social sciences (7)	14
				Master of health sciences (7)	15

3	Face-to-face	Public	3	Master of health care (7)	26
				Master of health sciences (7)	30
				No information available	15

4	Face-to-face	Public	1	Registered nurse (6)	40

5	Face-to-face	Private	2	Registered nurse (6)	22
				Registered nurse (6)	35

6	Remote	Public	2	Master of health care (7)	23
				Master of health care (7)	13

7	Face-to-face	Private	2	Registered nurse (6)	14
				Master of health care (7)	2

8	Face-to-face	Public	3	Registered nurse (6)	10
				Master's degree in physiotherapy (7)	12
				Registered nurse (6)	5

9	Face-to-face	Public	3	Master of health care (7)	22
				Master of health sciences (7)	6
				Master of health sciences (7)	6

Abbreviation: EQF = European qualification framework.

**Table 2:** An example of the interview guide.

Interview inquiry area	Questions	Probing questions	References
II	What kind of ethical issues have you encountered relating to:		
	- Comprehensive care of residents and cooperating with their family members	- Specify what makes the situation you describe an ethical issue. - Please provide practical examples from your work.	[6, 9, 10]

**Table 3:** Inductive content analysis.

Meaning unit	Condensed meaning unit	Grouped codes	Subtheme	Theme
Staff often visit me. My door is always open to make it as easy as possible for them to come and share their thoughts. They need a lot of support.	Staff need the NM's support to carry out their work and they often come to the NMs' office to ask for advice	Monitoring the implementation of good care and intervening in situations where it does not appear to be happening	Being present when staff are providing daily care to recognise ethically challenging situations	Ensuring residents' right to self-determination
We must carefully justify why we restrict residents from moving, so that we don't violate their rights to self-determination	Being able to justify why the right to self-determination is restricted	Ensuring justification for decisions regarding residents	Being aware of decisions relating to caring for the residents	
Some family members strictly forbade us from vaccinating residents. That is difficult, because you don't know the resident's views when they have lost their memory and ability to speak.	Separating the opinions of residents and family members is sometimes difficult	Ensuring that the opinions of residents and their family members are respected	Familiarity with residents and families to ensure person-centred care	

*Note:* Examples of meaning units, condensed meaning units, grouped codes, subthemes and themes.

**Table 4:** Ethical issues encountered by NMs in LTCS of older adults.

Theme	Subtheme
Ensuring residents' rights to self-determination	Being present when the staff are providing daily care, to recognise ethically challenging situations
	Being aware of the decisions related to the care residents receive
	Familiarity with residents and families to ensure person-centred care

Ethical decision-making regarding staff and procedures	Prioritising of responsibilities during daily work
	Treating staff equally but also meeting the individual needs of staff
	Maintaining ethical decision-making ability, even when instructions are incomplete or rapidly changing

Fulfilling ethical leadership, despite the conflicting roles on NMs in an organisation.	Receiving support in ethically challenging situations from a supervisor or senior managers
	Secondary tasks and misaligned support services leave less time for dealing with ethically important matters
	Obligations to the employer even against the NMs' own conscience

Defending ethics in LTCS on society level	Self-compassion in ethically burdensome situations
	Managing poorly resourced services and maintaining high standards
	Balancing between regulations and daily care needs

*Note:* Themes and subthemes.

## Data Availability

Because of the sensitive nature of the study, these data are not available to other researchers.
